# An asymmetric electrolyte to simultaneously meet contradictory requirements of anode and cathode

**DOI:** 10.1038/s41467-023-38492-8

**Published:** 2023-05-22

**Authors:** Shengmei Chen, Yiran Ying, Longtao Ma, Daming Zhu, Haitao Huang, Li Song, Chunyi Zhi

**Affiliations:** 1 Department of Materials Science and Engineering, City University of Hong Kong 83 Tat Chee Avenue, 999077 Kowloon, Hong Kong People’s Republic of China; 2 Department of Applied Physics and Research Institute for Smart Energy, The Hong Kong Polytechnic University, Hung Hom, 999077 Kowloon, Hong Kong People’s Republic of China; 3 Shanghai Synchrotron Radiation Facility, Shanghai Advanced Research Institute, Chinese Academy of Sciences, 201204 Shanghai, People’s Republic of China; 4 National Synchrotron Radiation Laboratory, CAS Center for Excellence in Nanoscience, University of Science and Technology of China, 230029 Hefei, People’s Republic of China; 5 Hong Kong Institute for Clean Energy, City University of Hong Kong, 999077 Kowloon, Hong Kong People’s Republic of China

**Keywords:** Batteries, Batteries

## Abstract

One of the major obstacles hindering the application of zinc metal batteries is the contradictory demands from the Zn metal anode and cathodes. At the anode side, water induces serious corrosion and dendrite growth, remarkably suppressing the reversibility of Zn plating/stripping. At the cathode side, water is essential because many cathode materials require both H^+^ and Zn^2+^ insertion/extraction to achieve a high capacity and long lifespan. Herein, an asymmetric design of inorganic solid-state electrolyte combined with hydrogel electrolyte is presented to simultaneously meet the as-mentioned contrary requirements. The inorganic solid-state electrolyte is toward the Zn anode to realize a dendrite-free and corrosion-free highly reversible Zn plating/stripping, and the hydrogel electrolyte enables consequent H^+^ and Zn^2+^ insertion/extraction at the cathode side for high performance. Therefore, there is no hydrogen and dendrite growth detected in cells with a super high-areal-capacity up to 10 mAh·cm^−2^ (Zn//Zn), ~5.5 mAh·cm^−2^ (Zn//MnO_2_) and ~7.2 mAh·cm^−2^ (Zn//V_2_O_5_). These Zn//MnO_2_ and Zn//V_2_O_5_ batteries show remarkable cycling stability over 1000 cycles with 92.4% and over 400 cycles with 90.5% initial capacity retained, respectively.

## Abstract (web summary)

While Zinc anodes are thermodynamically unstable in aqueous solutions, the protons (H+) from the water favor the cathodes of Zinc batteries. Here, the authors address this contradiction by designing an asymmetric electrolyte composed of an inorganic solid-state electrolyte and a hydrogel electrolyte.

## Introduction

Rechargeable aqueous Zn batteries stand out next-generation battery technologies due to their intrinsic safety, potentially low cost and sustainability^1–5^. A long-standing challenge to the practical implementation of Zn batteries as a typical aqueous system is the intricate contradiction between the functions of water at anode side and cathode side^6–8^. At the anode side, Zn corrosion, hydrogen evolution reaction (HER) and dendrite growth stem from the attraction between Zn^2+^ and water (forming [Zn(H_2_O)_6_]^2+^ coordination ions etc.) and uneven distribution of Zn deposites^9–12^. At the cathode side, however, H^+^ insertion& water “lubricant” effects are very important and sometimes essential to achieve a high capacity and superior cycling stability.

**Fig. 1:**
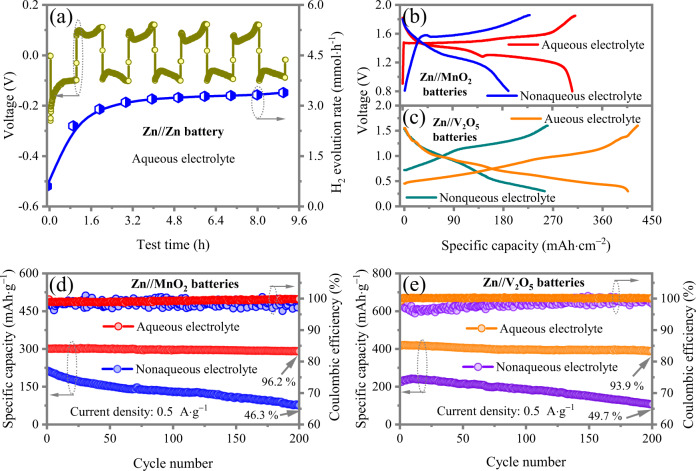
The evaluation of Zn//MnO_2_ and Zn//V_2_O_5_ full batteries using aqueous and non-aqueous electrolytes. **a** The hydrogen flux and Zn dendrites growth of Zn//Zn symmetric cell with aqueous electrolyte during deposition/dissolution process. The galvanostatic charge/discharge curves of **b** Zn//MnO_2_ batteries and **c** Zn//V_2_O_5_ batteries using aqueous and non-aqueous electrolytes. Cyclic stability of **d** Zn//MnO_2_ batteries and **e** Zn//V_2_O_5_ batteries using aqueous and non-aqueous electrolytes.

As shown in Fig. 1a, the Zn//Zn symmetric cell evolutes 2.4 mmol·h^−1^·cm^−2^ hydrogen in the first cycle and the rate of hydrogen evolution increases to 3.38 mmol·h^−1^·cm^−2^ after 9 h test at 10 mA·cm^−2^ with 10 mAh·cm^−2^ of Zn reversibly cycled, confirmed by in situ battery-gas chromatography-mass spectrometry (GC-MS) system. The anabatic side hydrogen evolution comes from the increase of contact area between Zn metal and electrolyte due to uneven distribution of Zn deposition. The HER and Zn dendrites in aqueous Zn batteries are originated from the water decomposition during Zn deposition process^13–15^. Consequently, large excesses of Zn and electrolyte are required to replenish anode and water consumption during cycling, which decreases the overall energy density and cycle life of the batteries, and even induces short-circuit and battery failure. Extensive attentions have been paid to addressing this challenge, such as high concentration electrolytes^16,17^, organic electrolytes^18,19^, organic solvent additives^6^, hydrogel electrolyte^20,21^, polymer solid electrolyte^22^, inorganic solid electrolytes^23^, surface coating^24,25^, alloy metal electrode^26,27^ and 3D structured current collectors^26,28^. Nevertheless, these strategies can only alleviate but do not thoroughly prevent Zn degradation as the Zn/water interface is fundamentally thermodynamically unstable^29^.

On the other hand, most cathodes of Zn-ion batteries such as Mn- and V-based oxides requires both Zn^2+^ and H^+^ insertion/extraction during charge/discharge process for full utilization of electrode materials, reduction of Zn^2+^ insertion/extraction energy and high-performance output^9,30–32^. We use 1 M Zn(OTf)_2_ in distilled water and 1 M Zn(OTf)_2_ in trimethyl phosphate (TMP) as aqueous and non-aqueous electrolytes, respectively. For the typical Zn//MnO_2_ batteries, two discharge plateaus corresponding two-step reactions are demonstrated and a capacity of ~300 mAh·g^−1^ close to theoretical value is delivered with an aqueous electrolyte. Whereas only one lower discharge plateau and the specific capacity of 191.1 mAh·g^−1^ are delivered in a non-aqueous electrolyte (Fig. 1b). Similarly, as shown in Fig. 1c, only a capacity of 255.1 mAh·g^−1^ is delivered in the Zn//V_2_O_5_ battery employing a non-aqueous electrolyte, much lower than that using aqueous electrolyte (408.3 mAh·g^−1^). Furthermore, water can also significantly enhance the Zn ions diffusion in cathode owing to the effectively reduced solid-state diffusion barriers^33^. Owing to the “lubricant” effect of water molecular, the Zn//MnO_2_ battery retains 96.2% initial capacity after 200 cycles. In contrast, the retention is only 46.3% when non-aqueous electrolyte is used. (Fig. 1d). For the Zn//V_2_O_5_ batteries, the capacity decays to 49.7% initial capacity after only 200 cycles with non-aqueous electrolyte, while the battery gives 93.9% capacity retention with an aqueous electrolyte (Fig. 1e). Hence, water as a source of H^+^ and lubricant is essential for cathode reactions of Zn batteries in most cases.

Here, for the first time, we design an asymmetric electrolyte to not only solve the issues of Zn instability and Zn dendrite growth, but also maintain the electrochemical performance of aqueous Zn batteries intact. The asymmetric electrolyte consists of two functionalized layers. On Zn metal side, the highly Zn^2+^ conductive inorganic solid-state electrolyte is designed to achieve dendrite-free and hydrogen-free Zn deposition/dissolution. On the cathode side, a polyacrylamide (PAM) hydrogel electrolyte contains aqueous solution to meet additional H^+^ insertion/extraction and facilitate the Zn^2+^ insertion/extraction. In this way, the dendrite-free and side-reactions-free aqueous Zn//MnO_2_ and Zn//V_2_O_5_ batteries with high-areal capacity (>5 mAh·cm^−2^) and ultralong-cycle life are developed and ampere hour-level (~1100 mAh) Zn//MnO_2_ battery is demonstrated to present the effective tactic of asymmetric electrolyte design.

## Results and discussion

### The design of asymmetric electrolytes

We design an asymmetric electrolyte to meet the contradictory requirements of Zn-metal anode and cathode concurrently. Specifically, the asymmetric electrolyte consists of two functionalized layers depicted as follows (Supplementary Fig. 1): (i) on the Zn metal side, we developed a highly Zn^2+^ conductive inorganic solid-state electrolyte (SSE) based on two-dimensional (2D) porphyrin paddlewheel framework (PPFs) metal-organic framework sheets. The PPFs-derived SSEs (PPF-SSEs) can effectively guide Zn uniform deposition, isolate Zn electrode from bulk aqueous electrolyte and force the Zn(H_2_O)_6_^2+^ ions desolvation to achieve dendrite-free and hydrogen-free Zn battery cycles. (ii) on the cathode side, a PAM hydrogel electrolyte with super-absorbency of aqueous solution provides enough H^+^ as additional charge carrier and water molecule as “lubricant” agent facilitating the Zn^2+^ insertion/extraction. As a whole, the asymmetric design not only solves the issues of Zn instability and Zn dendrite growth, but also maintains the electrochemical performance of aqueous Zn batteries intact.

**Fig. 2:**
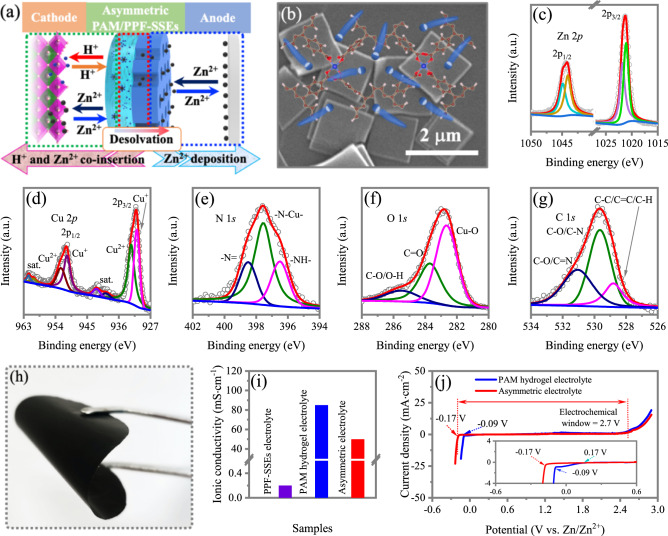
The preparation and characterization of asymmetric electrolyte. **a** Schematic illustration of asymmetric electrolyte design (including one-layer PPFs-SSEs and one-layer PAM hydrogel electrolyte). **b** SEM image of the PPFs-SSEs. The inset is schematic illustration of crystal structure of the 2D PPF-SSEs. Zn^2+^ ions are highlighted by blue balls. High-resolution XPS spectra of **c** Zn 2*p*, **d** Cu 2*p*, **e** N 1 *s*, **f** O 2 *s* and **g** C 1 *s* in the PPFs-SSEs. **h** Optical image of PAM/PPF-SSEs asymmetric electrolyte films. **i** Ionic conductivity of PPF-SSEs, hydrogel electrolyte and the asymmetric electrolyte. **j** Electrochemical window of the designed asymmetric and hydrogel electrolyte measured by LSV at 5 mV·s^−1^. The inset is the enlarged view of Zn^2+^ deposition curves.

The PPF-SSEs are prepared by ion instillation method. Firstly, the 2D PPFs square-like sheets with lateral size of ~2.5 μm is prepared (Supplementary Fig. 2a) and the sharp X-ray diffraction (XRD) peaks of PPF nanosheets indicate a tetragonal structure with good crystallinity (Supplementary Fig. 2b)^34,35^. The PPF-SSEs films are obtained by instilling Zn^2+^ into PPF channels by repeatedly immersing PPF nanosheets in 0.5 M Zn(OTf)_2_ trimethyl phosphate (TMP) solution under subatmospheric pressure condition and evaporates solvent at 120 ^o^C under vacuum condition for several cycles to get enough Zn^2+^ ions in PPF channels. As shown in Supplementary Fig. 3, **t**he Brunauer-Emmett-Teller (BET) surface area and pore volume of PPFs are calculated to be 808 m^2^·g^−1^ and 0.98 cm^3^·g^−1^, respectively, which dropped to 362 m^2^·g^−1^ and 0.41 cm^3^·g^−1^ for the PPF-SSEs as a result of Zn^2+^ uptake. The PPF-SSEs film is fabricated by blade coating PPF-SSEs powders on a glass substrate, tearing off from the glass substrate and compressing to decrease possible existing inter-particle pores. Finally, the asymmetric electrolyte is obtained by in situ polymerizing partial-polymerized acrylamide sol films with ultra-high viscosity on one side of the PPF-SSEs film (Fig. 2a). It is noted that after the ion instillation and drying process, the PPF-SSEs still show well-crystalline structure (Fig. S2b) and maintain the square-like nanosheet morphology and uniform lateral size of 2.5 μm (Fig. 2b), manifesting the excellent stability of the MOF structure. The PPF-SSEs chemistry is investigated using X-ray photoelectron spectroscopy (XPS). Before test, the PPF-SSEs is etched by Ar^+^ for 5 min to scavenge the surface residual. The apparent presence of high-resolution Zn 2*p* spectra is observed, and the percentage of Zn^2+^ ions reach to 32 wt.% (Fig. 2c), indicating the successful insertion of Zn^2+^ into the PPFs channels. Furthermore, as shown in Supplementary Fig. 4 and Supplementary Table 1, compared with Zn(OTf)_2_, the coordination number of PPF-SSEs is increased from 2.9 to 3.7, and the bond length shortens from 2.07 to 1.96 Å, which manifests strong bonding effects of Zn^2+^ in the PPF-SSEs and interactions of Zn^2+^ with the PPF-SSEs. In addition, the appearance of Zn-Zn coordination shell demonstrates the Zn-Zn binding action in the crowded PPF-SSEs channels, which further attests the successful insertion of Zn^2+^ in PPF-SSEs. The Cu 2*p* reveals the co-existence of two Cu valence state of Cu^2+^ and Cu^+^ (Fig. 2d). The Cu metal species bond with N and O species with Cu-N and Cu-O bonds can be confirmed by N 1*s* (Fig. 2e) and O 1*s* spectra (Fig. 2f). The Cu-O bonds can be further confirmed by the Fourier-transform infrared spectroscopy (FT-IR) of Cu-O-C stretching vibrations at 1539. 27 and 1606.25 cm^−1^ (Supplementary Fig. 5). The C 1*s* core level peaks can be resolved into three components of C-C/C=C/C-H, C-O/C-N and C-O/C=N bonds, respectively (Fig. 2g). Meanwhile, strong absorption bands in the region from 1418 to 1677 cm^−1^ in FT-IR spectra, assigned to carboxyl and pyrrole stretching vibrations within the 5, 10, 15, 20-tetrakis(4-carboxyl-phenyl)-porphyrin (TCPP) ligands. The above results indicated the structure of PPFs is completely maintained and no Zn composites are formed during the prepare process of PPF-SSEs.

To fabricate asymmetric electrolyte (PPF-SSEs/PAM), the PPF-SSEs film is firstly prepared and PAM hydrogel layer is in situ polymerized on one side of PPF-SSEs, in which the affluent hydroxyl groups PPFs surface supply plentiful grafting sites for the PAM chains. The photograph of the as-prepared asymmetric electrolyte is shown in Fig. 2h and Supplementary Fig. 6. The asymmetric electrolyte with a thickness of 210 µm, shows a smooth surface with dark color and decent flexibility. The ionic conductivity is one of the most significant indicators. The PPF-SSEs shows a relatively low ionic conductivity of 0.21 mS·cm^−1^, compared to the that of PAM hydrogel electrolyte (84.79 mS·cm^−1^) at room temperature. Whereas, after assembling PPF-SSEs with PAM hydrogel electrolyte, the asymmetric electrolyte delivers a high ionic conductivity of 50.86 mS·cm^−1^ (Fig. 2i, Supplementary Fig. 7), which is ascribed to the enhanced activity of Zn^2+^ ions in the channel of PPFs after absorbing trace 10.9 wt% water (vs. PPF-SSE) from the surface of PAM hydrogel electrolyte (Supplementary Fig. 8a)^23^. The traced water with “lubricant” effect is utilized to solvated Zn^2+^ in large cavities of the electrical insulated MOFs to promote Zn^2+^ transfer (Supplementary Fig. 8b). Furthermore, the electrochemical stability of the asymmetric electrolyte and PAM hydrogel electrolyte are evaluated on a non-active Ti electrode using linear scan voltammetry (LSV) (Fig. 2j). The oxidation onset potentials of the asymmetric electrolyte are reasonably close to that of PAM hydrogel. Whereas, for the anode side, there is no water reduction observed and Zn begins to deposition at −0.17 V (inset of Fig. 2j), in contrast to water reduction at 0.17 V and Zn deposition at −0.07 V for the PAM hydrogel electrolyte. The negatively shift potential for Zn deposition with asymmetric electrolyte originates from energy dissipation for desolvation process of Zn^2+^ ion from Zn(H_2_O)_6_^2+^ coordination ion and high activation energy of Zn^2+^ ions transfer in the PPF-SSEs channels. Hence, the developed asymmetric electrolyte can immensely eliminate side reactions of Zn electrode in water.

### Desolvation effect of the PPFs-SSEs

**Fig. 3:**
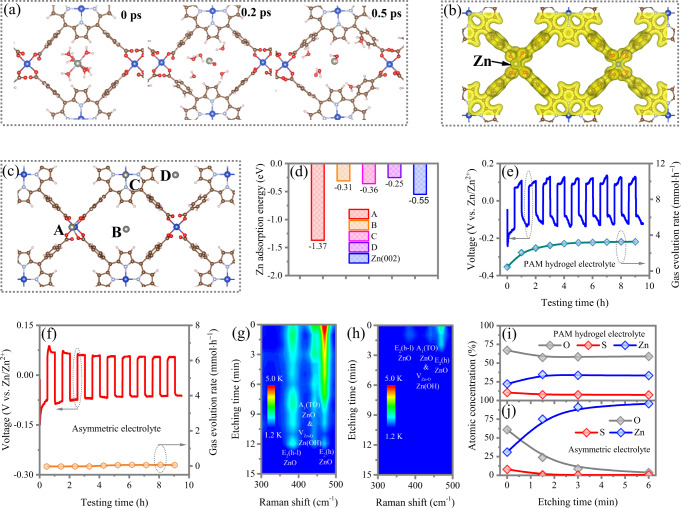
Theoretical simulation and experimental analysis of the desolation effect for the PPFs-derived solid electrolyte layer. Theoretical simulation and experimental analysis of the desolation effect for the PPFs-derived solid electrolyte layer. **a** AIMD simulation of the desolvation process at the PPFs surface at 300 K at 0, 0.2, 0.5 ps. **b** The charge density of PPFs with Zn^2+^ adsorption. **c** The possible adsorption sites of Zn^2+^ on PPFs surface. **d** Adsorption energy of Zn^2+^ on different adsorption sites and Zn (0002) surface. The hydrogen flux of Zn//Zn symmetric cells using **e** PAM hydrogel electrolyte and **f** asymmetric electrolyte. Raman spectra of Zn electrode with **g** hydrogel electrolyte and **h** asymmetric electrolyte. The element analysis of Zn metal with different etching time by XPS analysis **i** hydrogel electrolyte and **j** asymmetric electrolyte.

The desolvation effect of PPF-SSEs are scrutinized by density functional theory (DFT) calculation and ab initio molecular dynamics (AIMD) simulation. In the aqueous electrolyte, the Zn^2+^ ions bonds with six water molecules forming Zn(H_2_O)_6_^2+^ coordination ions, which will experience a desolvation process before being reduced to Zn. At the same time, the coordinated Zn(H_2_O)_6_^2+^ ion would desolventize on the Zn surface. In this process, the H_2_O molecules directly encounter the Zn metal anode, inevitably leading to the corrosion of Zn electrode and HER^25,36^. Whereas, for the PPF-SSEs, the DFT results show that the Zn(H_2_O)_6_^2^ can be strongly adsorbed on PPFs with the adsorption energy value of −3.89 eV. The AIMD simulation of PPFs with Zn(H_2_O)_6_^2+^ adsorbed on surface is further performed to investigate the desolvation process at the temperature of 300 K. A clear tendency of Zn(H_2_O)_6_^2^ desolvation process is observed after 0.2 and 0.5 ps (Fig. 3a), which is further confirmed by the decreasing trend of the total energy of the system (Supplementary Fig. 9). The adsorption energies of H_2_O and Zn^2+^ in channels of PPFs are calculated to be −0.76 and −0.31 eV, respectively, proving that PPFs has a strong interaction with H_2_O molecules, enabling an enhanced desolvation effect. The Raman and FT-IR measurements are further conducted to characterize desolvation of water from Zn in the PPF-SSEs. As shown in Supplementary Fig. 10a, the PAM hydrogel electrolyte exhibits a broad Raman band in range of 3000-3800 cm^−1^ (O-H stretching vibration mode), which is associated with strong hydrogen-bonding environmental in water cluster. This broad band substantially weakens in intensity and shifts to a low frequency at the interface of PAM/PPF-SSEs, suggesting the decreased amount of Zn[H_2_O)_6_]^2+^ and a changed solvation structure of Zn^2+^. In comparison, the peak representing O-H stretching vibration vanishes in the PPF-SSEs, indicating its desolvation effect. Similar behavior can be inferred from FT-IR spectra (Supplementary Fig. 10b). Both the characteristic “intermediate water” (3350 cm^−1^) and “multimer water” (3450 cm^−1^) related broad O-H stretching vibration can be observed in the PAM hydrogel electrolyte but disappear at anode side of the PPF-SSEs. Both Raman and FT-IR results corroborates that bulk water molecules nearly do not exist at anode side of the PPF-SSEs, which is ascribed to effective desolvation effect of PPF-SSEs.

Furthermore, the charge density and charge density difference distribution in Fig. 3b, Supplementary Fig. 11 represent significant charge transfer between Zn^2+^ and PPFs (the number of charge transfer from Zn to PPFs is estimated to be 0.86), together with the larger adsorption energy of Zn^2+^ on surface of PPFs (−1.37 eV) compared with Zn(002) (−0.55 eV) (Fig. 3c, d), which reveals the strong Zn^2+^ immobilization effect of PPFs. The immobilization effect promotes to build a uniform super-saturated electrolyte on the front surface of PPFs, which can further reduce the activity of water molecular. The strong desolvation and immobilization effects significantly prohibit the process of water-induced Zn corrosion and HER side reactions.

In experiment, the direct proofs for the suppressed side reactions enabled by PPF-SSEs are obtained from in situ monitoring hydrogen flux during Zn reversible deposition/dissolution process and detecting component evolution of cycled Zn anode harvested from Zn//Zn cells after ten cycles. It should be mentioned that a PPF-SSE/PAM/PPF-SSE sandwich electrolyte in Zn//Zn cells for certification of desolvation effect of PPF-SSEs. As shown in Fig. 3e, the hydrogen evolution rate during Zn//Zn cell at 10 mA·cm^−2^ with 10 mAh·cm^−2^ using PAM hydrogel electrolyte is 1.96 mmol·h^−1^·cm^−2^ at first cycle and increases to 3.39 mmol·h^−1^·cm^−2^ at the ninth cycle. The hydrogen evolved during the resting process (0.42 mmol·h^−1^·cm^−2^) is ascribed to chemical oxidation stemming from thermodynamic instability of Zn in aqueous system. Encouragingly, there is almost no hydrogen detected in Zn//Zn cell using PPF-SSEs/PAM/PPF-SSEs electrolyte, revealing the completely suppressed HER using our asymmetric electrolyte design (Fig. 3f). The direct evidence for the eliminated hydrogen evolution enabled by the PPF-SSE is achieved from in situ optical visualization observations of Zn deposition. Abundant gas bubbles are observed even 5 min after the commencement of deposition with an applied constant current density of 10 mA·cm^−2^ for the bare Zn foil (Supplementary Fig. 12a). In comparison, for the Zn electrode with the PPF-SSE under the same condition, there are no any bubbles observed even after over 25 min (Supplementary Fig. 12b).

To systematically examine the component evolution of the Zn anode after cycling test, we firstly conduct XRD to detect the side-effect of decomposition on the Zn surface. The distinct side-product of Zn_4_SO_4_(OH)_6_·H_2_O is observed in cycled Zn anode harvest from Zn//Zn cell with PAM hydrogel electrolyte, whereas there is almost no side-product detected employing the asymmetric electrolyte (Supplementary Fig. 13). We further employ space-resolution operando Raman spectra and XPS, coupled with ion sputtering technique to analyze the component of accumulated products on Zn anode. Three evident phonon models of ZnO crystals referred to E_2_(h) (467 cm^−1^), A1(TO) (442 cm^−1^) and E_2_(h-l) (385 cm^−1^) models revealing the growth of ZnO crystal in Zn anode surface without preferred orientation^37^. The formation of Zn(OH)_2_ species is confirmed by the vibration of Zn-O centered at 438 cm^−1^ and the existence of Zn_4_SO_4_(OH)_6_·H_2_O can be further identified by the appearance of sulfur element in XPS spectra. With the increase of detection depth, the typical peaks of ZnO and Zn(OH)_2_ maintain in Raman spectra (Fig. 3g) and the content of oxygen and sulfur almost keep unchanged in XPS spectra (Fig. 3h, Supplementary Fig. 14) for the Zn anode with the PAM hydrogel electrolyte, indicating that thick passivation layer and corrosion reaction occupies the whole Zn electrode from the surface to the inner part. In a sharp comparison, for the Zn foil harvesting from cells with asymmetric electrolyte, the intensity of ZnO and Zn(OH)_2_ peaks in Raman spectra is very weak and the peaks quickly disappears only after 2 min etching operation (Fig. 3i, Supplementary Fig. 15). Meanwhile, the content of oxygen and sulfur is significantly low and rapidly decrease to zero in XPS spectra after 3 min etching operation (Fig. 3j). The above results reveal a shallow passivation film on Zn anode, which can be ascribed to the inevitable minor water molecule releasing during the battery working.

### Electrochemical behaviors of Zn electrodeposition

**Fig. 4:**
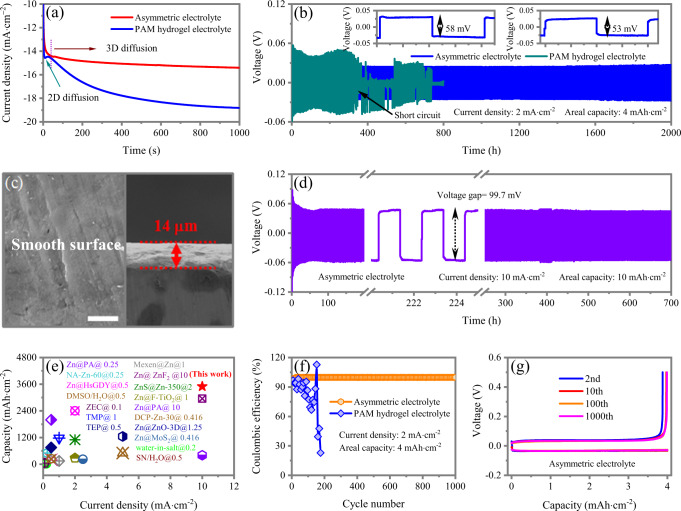
The electrochemical performance of Zn//Zn symmetric and Zn//Cu asymmetric cells using conventional hydrogel electrolyte and the designed asymmetric electrolyte. **a** Chronoamperometry tests of Zn metal with hydrogel and asymmetric electrolytes at −150 mV overpotential. **b** Comparison of cycling stability of Zn//Zn symmetric cells using hydrogel electrolyte and the designed asymmetric electrolyte at 2 mA·cm^−2^ with a capacity of 4 mAh·cm^−2^. The inset is the voltage profiles of symmetric Zn//Zn cell at 5th and 1000th cycles, respectively using the asymmetric electrolyte. **c** SEM images and cross-section SEM images of Zn anode after cycling using the asymmetric electrolyte. Scale bar: 5 μm. **d** Cycling stability of the Zn//Zn symmetric cells using asymmetric electrolyte at 10 mA·cm^−2^ with a capacity of 10 mAh·cm^−2^. **e** Comparison of cumulative capacity recycled and current density between this work and the previously reported ones. Different works are distinguished by color. **f** Coulombic efficiency of Zn plating/stripping in Zn//Cu cell at 2 mA·cm^−2^ with a capacity of 4 mAh·cm^−2^. **g** Voltage profiles of Zn//Cu cell using asymmetric electrolyte at the 2nd, 10th, 100th, 1000th cycles.

The Zn deposition behaviors with the developed asymmetric electrolyte and compared PAM hydrogel electrolyte are examined by Chronoamperometry (CA). Under constant potential, the change of deposition current with times can sensitively represent the nucleation and surface change^38,39^. With PAM hydrogel electrolyte, the deposition current progressively increases within 1000 s, revealing a rampant 2D diffusion behavior and thus uneven Zn deposition (Fig. 4a). In deposition process, the desolvated Zn^2+^ ions are absorbed on Zn metal surface and diffuses laterally along the surface to find preferred sites for reducing surface energy. Thus, the adsorbed Zn^2+^ ions tend to form aggregations and further grow on the tip, thus accelerating dendrite growth. In sharp contrast, the nucleation and 2D diffusion occurs within 50 s with the asymmetric electrolyte and steady 3D diffusion proceeds with a relative constant current density of −15.3 mA·cm^−2^, deducing a homogenization of Zn^2+^ distribution and normalization, and thus giving homogeneous nucleation sites for subsequent Zn smooth deposition. The real capacity of Zn deposition is evaluated by calculating the deposited capacity and the weight of Zn anode dissolution with the Zn//Cu cell. As shown in Supplementary Fig. 16, specific capacities of 809.72 and 793.65 mAh·g^−1^ are achieved at 2 and 10 mAh·cm^−2^, respectively. The small loss of capacity originates from the absorption of Zn^2+^ ions in the channels of the PPF-SSEs.

The electrochemical stability of Zn anode is scrutinized by galvanostatic cycling of symmetric Zn//Zn cells. Under constant current density of 2 mA·cm^−2^ with areal capacity of 4 mAh·cm^−2^, the Zn//Zn cell using PAM hydrogel electrolyte exhibits a large overpotential up to 55 mV and the potential profile of becomes substantially fluctuated after only 300 h, indicating poor interfacial stability due to the formation of Zn_4_SO_4_(OH)_6_·H_2_O, ZnO, Zn(OH)_2_ by-products layer caused by water-induced corrosion (Fig. 4b). Meanwhile, during cycling, the highly porous/mossy structure of deposited Zn (30 µm) exhibits and plenty of dendrites grow on Zn surface (Supplementary Fig. 17), which will partly penetrate the hydrogel electrolyte. Zn dendrite penetration through hydrogel is considered as the major reasons for short-life Zn anodes due to the high Young’s modulus of Zn (*E*_Zn_ = 108 Gpa). Before the ultimate short-circuit, the cell presents a signal of cell short-circuit by a sudden voltage drop and continuous potential fluctuation. Inversely, Zn deposits from asymmetric electrolyte is smooth and compact, as supported by a thin deposited Zn layer (14 µm) in cross-sectional imaging (Fig. 4c). The uniform Zn deposition enables the Zn//Zn cells stably operate over 2000 h with a low overpotential of 29 mV and without potential fluctuation, which infers a significantly strengthened interfacial stability on Zn anode. Even at a high current density of 10 mA·cm^−2^ with high areal capacity of 10 mAh·cm^−2^, Zn//Zn cell with asymmetric electrolyte can works steadily for 700 h with only 50 mV overpotential and without apparent irreversible voltage observed (Fig. 4d), which outperforms most of its counterparts with dendrite precaution-oriented and HER-suppressed designs (Fig. 4e).

The reversibility of Zn electrode in the asymmetric electrolyte and PAM hydrogel electrolyte is further compared in Zn//Cu cells at a current density of 2 mA·cm^−2^ with a high areal capacity of 4 mAh·cm^−2^. The Zn plating/stripping in hydrogel electrolyte demonstrate a low Coulombic efficiency of 90.3% in initial several cycles, declining to 82.5% at 150th cycles and failing after 170 cycles (Supplementary Fig. 18). In a sharp contrast, with the asymmetric electrolyte, the Coulombic efficiency is 98.3% in 2 cycles and increases to 99.9% within 10 cycles, averaging to 99.9% across 1000 cycles (Fig. 4f, g). The outstanding reversibility of Zn deposition/dissolution is attributed to asymmetric electrolyte design, in which PPF-SSEs function as effective desolvation layers to avoid water molecules from PAM hydrogel electrolyte reacting with Zn electrode, together with its fast Zn^2+^ ions diffusion of PPF-SSEs.

### Electrochemical performance of high-areal-capacity Zn ion full cells

To further examine the smart strategy of the asymmetric electrolyte for both anode and cathode, high-areal-capacity pouch-type Zn//MnO_2_ batteries are assembled by employing a Zn metal anode, δ-MnO_2_ cathode (Supplementary Fig. 19) necessarily experiences the deposition/dissolution reaction of Mn^2+^/MnO_2_ to achieve a capacity of 306 mAh·g^−1^. Most previous work adopted a very low areal capacity (always <0.5 mAh·cm^−2^) to hide the Zn anode problems for an optimized stability. Here, a high-areal capacity of ~5.5 mAh·cm^−2^ was directly adopted for the fully pouch-type cells, in which the 2 cm$\,\times$ 2 cm $\times$ 2 μm Zn foil with weight of 0.058 g and the same dimension of cathode are used to assemble high-areal capacity pouch cell. The utilization of Zn foil is ~42% and ~0.024 g Zn is cycled during each charge/discharge process.

**Fig. 5:**
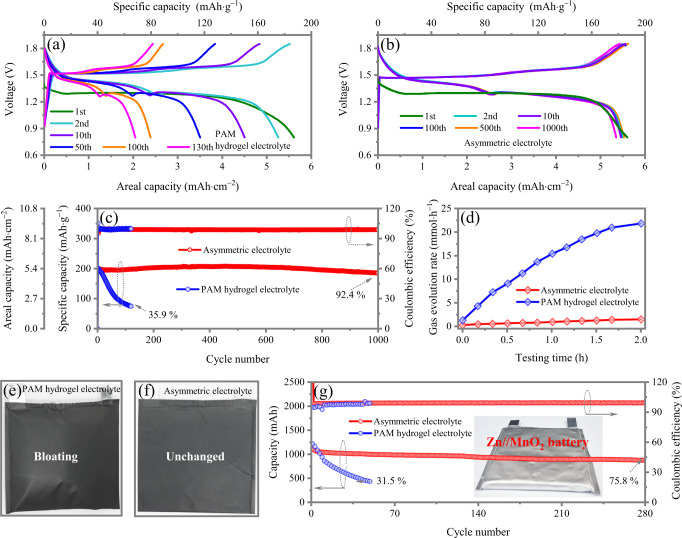
The electrochemical performance of high-areal-capacity Zn//MnO_2_ full cell using conventional hydrogel electrolyte and the designed asymmetric electrolyte. Galvanostatic charge/discharge profiles of Zn//MnO_2_ full cell using **a** hydrogel electrolyte at 1st, 2nd, 10th, 100th, 130th, and **b** asymmetric electrolyte at 1st, 2nd, 10th, 100th, 500th, 1000th cycles. **c** Corresponding cyclic stability of Zn//MnO_2_ full cells using asymmetric electrolyte and hydrogel electrolyte. **d** In situ monitoring of hydrogen flux on a high-areal-capacity Zn//MnO_2_ full cell using with PAM hydrogel and asymmetric electrolytes during charging process. Optical photos of a Zn//MnO_2_ with a size of 2×2 cm^2^ using **e** PAM hydrogel and **f** asymmetric electrolytes after cycling tests. **g** Cyclic stability of a ~1100 mAh large-capacity Zn//MnO_2_ with the asymmetric electrolyte.

The Zn//MnO_2_ batteries delivers a high specific capacity of ~195 mAh·g^−1^ at high rate of 5C with the asymmetric electrolyte, which is the same with that with the PAM hydrogel electrolyte. This reveals that the hydrogel side of the asymmetric electrolyte works well to release capacity of MnO_2_ by providing an H_2_O environment for H^+^ contribution. On the other hand, compared with Zn//MnO_2_ batteries operating only 130 cycles (35.9% capacity retention) with the PAM hydrogel electrolyte, (Fig. 5a), with asymmetric electrolyte, the Zn//MnO_2_ batteries exhibit an outstanding cyclability of 1000 cycles with 92.4% initial capacity retained at nearly 100% Coulombic efficiency (Fig. 5b, c). This confirms the PPF side of the asymmetric electrolyte works well to provide a highly reversible Zn plating/stripping chemistry. The high rate comes from the high conductivity of solvated [Zn(H_2_O)_6_]^2+^ in large cavities of the electrical insulated MOFs. The desolvation effect of hydrophilic microporous MOF can prevent H_2_O from reaching the Zn anode. It not only inhibits the process of water-induced Zn corrosion but also benefits the kinetics of Zn^2+^ plating/stripping. Compared with Zn anode using PAM hydrogel electrolyte displaying porous structure and thin platelets, asymmetric electrolyte enables smooth and intact Zn surfaces to be recovered from prolonged cycling (Supplementary Fig. 20a, b).

To accurately quantify the hydrogen produced in Zn//MnO_2_ full batteries operation, the 2×2 cm^2^ pouch-type cells are constructed and utilized to monitor the hydrogen flux. The detailed tests reveal that with hydrogel electrolyte, the hydrogen flux of Zn//MnO_2_ cell increases and reaches a maximal value of 21.8 mmol·h^−1^ at the fully charged state. Whereas there is almost no hydrogen detected in cell with asymmetric electrolyte (Fig. 5d). Meanwhile, as depicted in Fig. 5e, f, visually the pouch cell with hydrogel electrolyte is remarkably swollen, while there is no change of Zn//MnO_2_ cell with the asymmetric electrolyte. The significantly suppressed HER and dendrite-free properties during cycling manifests the triumphant restraint of side reactions by the asymmetric electrolyte, which thus promotes the reversibility of electrochemical plating/stripping of Zn anode.

The outstanding performance of high-areal-capacity Zn//MnO_2_ pouch cell inspires us to construct laminated multilayer-electrodes pouch-type batteries. The battery configuration consists of four whole sets of anode-separator-cathode stacks with a size of 7.5 × 8.5 cm^2^, which is fabricated in ambient air environment without complicated procedures and protections (Supplementary Table 2). Following the industrialized route, the sufficient rolling step would largely reduce the cathode porosity, bringing remarkable improvement in area weight loading and the ratio of active materials/electrolyte. As shown in Fig. 5g, the ~1100 mAh Zn//MnO_2_ battery with asymmetric electrolyte exhibits prominent cyclic stability with 75.8% initial capacity retained after 280 cycles. In comparison, the Zn//MnO_2_ battery with PAM hydrogel electrolyte suddenly decays to 31.5% only after 50 cycles. The results demonstrate excellent largescale and practical use of our strategies.

**Fig. 6:**
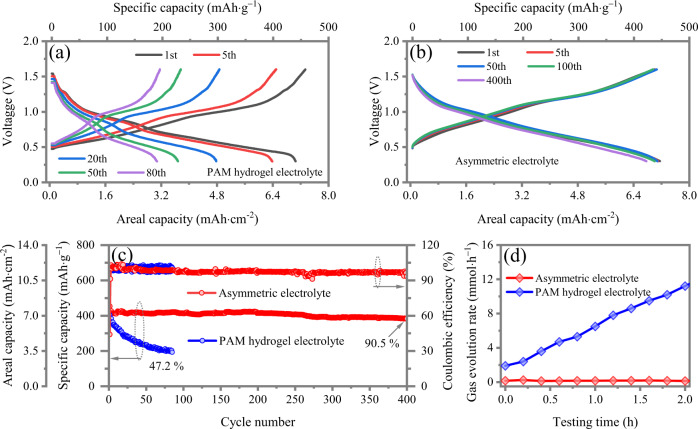
The electrochemical performance of high-areal-capacity Zn//V_2_O_5_ full cell using conventional hydrogel electrolyte and the designed asymmetric electrolyte. Galvanostatic charge/discharge profiles of **a** Zn//V_2_O_5_ full cell using asymmetric electrolyte at 1st, 5th, 20th, 50th and 80th cycles, and **b** Zn//V_2_O_5_ full cell using asymmetric electrolyte at 1st, 5th, 50th, 100th, and 400th cycles. **c** Corresponding cyclic stability of Zn//V_2_O_5_ full cells using asymmetric electrolyte and hydrogel electrolyte. **d** The hydrogen flux of Zn//V_2_O_5_ batteries using PAM hydrogel and asymmetric electrolytes during charging process.

To further demonstrate the universality of asymmetric electrolyte to aqueous metal-ion batteries, vanadium oxide bronze (V_2_O_5_) (Supplementary Fig. 21) with a high theoretical capacity of 589 mAh·g^−1^ is also used as cathode to couple with the Zn electrode. Similarly, the capacity of Zn//V_2_O_5_ battery with PAM hydrogel electrolyte containing 2 M ZnSO_4_ solution quickly fades to 47.2% initial capacity after only 85 cycles (Fig. 6a). In contrast, with asymmetric electrolyte, the battery exhibits excellent long-term cycling stability (90.5% initial capacity after 400 cycles at 4 A·g^−1^) (Fig. 6b, c). Compared with Zn//V_2_O_5_ battery using PAM hydrogel electrolyte with 13.3 mmol·h^−1^ at fully charged state and serious dendrite growth (Fig. 6d), the battery with asymmetric electrolyte also preserves hydrogen-free and dendrite-free properties.

In summary, to solve the fundamental contradiction of thermodynamically unstable Zn anode in H_2_O and H^+^/H_2_O favored cathode of Zn batteries, we propose an asymmetric electrolyte consisting of inorganic solid-state and hydrogel layers for Zn batteries to fulfill the requirement of both Zn anode and cathode. In the assembled batteries, Zn anode faces the solid-state PPF side of the asymmetric electrolyte, achieving a dendrite-free& hydrogen-free high reversibility; Cathodes face the hydrogel side of the asymmetric electrolyte, realizing their full capacity by utilizing H_2_O and H^+^ in the hydrogel. The developed electrolyte exhibits outstanding performance in a symmetric battery. More importantly, at full cell level, a ~5.5 mAh·cm^−2^ high-areal-capacity Zn//MnO_2_ battery utilizing asymmetric electrolyte delivers a high-capacity retention up to 92.4% after 1000 cycles with an extra-high Coulombic efficiency of 99.9% per cycle. In addition, a ~7.2 mAh·cm^−2^ high-areal-capacity Zn//V_2_O_5_ battery delivers a high-capacity retention reaching 90.5% after 400 cycles with an extra-high Coulombic efficiency exceeding 99.9% per cycle. This work will provide a smart solution to construct bi-functional electrolyte to meet simultaneously contradictory requirements of anode and cathode of Zn batteries. The demonstrated large cells represent a solid progress towards practical application of Zn batteries.

## Methods

### Synthesis of porphyrin paddlewheel framework (PPF) nanosheets

The PPF nanosheets are synthesized according to previous work^40^. In details, 0.015 mmol Cu(NO_3_)_2_·6H_2_O, 0.01 mmol 4, 4’-bipyridine (BPY) and 10 mg Polyvinylpyrrolidone (PVP, wt. 40,000) are dissolved in 6 mL of mixture of N, N-Dimethylformamide (DMF) and ethanol (*v*:*v* = 3:1) in a 10 mL vial. The 0.005 mmol 5, 10, 15, 20-tetrakis(4-carboxyl-phenyl)-porphyrin (TCPP) dissolved in 2 mL of the mixture of N, N-Dimethylformamide (DMF) and ethanol (*v*:*v* = 3:1) is added above solution and mixed under ultrasonic condition for 1 h. After that, the vial is sealed and heated to 100 ^o^C, and kept at 100 ^o^C for 24 h. The PPF nanosheets are obtained by centrifuging and washed by DMF and ethanol.

### Fabrication of PPFs-derived solid-state electrolytes (PPF-SSEs) powder

The PPF-SSEs are fabricated by ion diffusion. 0.5 g PPF nanosheets are dispersed in 50 mL 0.5 M Zn(OTf)_2_ trimethyl phosphate (TMP) solution under subatmospheric pressure condition and evaporates TMP solvent at 120 ^o^C under vacuum condition for several times to get enough Zn^2+^ in PPF channels. After that, the PPF-SSEs are obtained by washing residual Zn(OTf)_2_ on the surface of PPFs with TMP and activated at 150 ^o^C in vacuum for 12 h.

### Preparation of PPF-SSEs/PAM hydrogel asymmetric electrolyte

Firstly, PPF-SSEs slurry is obtained by mixing 95 wt% PPF-SSEs with 5 wt% Polytetrafluoroethylene (PTFE) in an absolute ethanol solution. After thoroughly stirring for 30 min, the sticky PPF-SSEs slurries are then uniformly spread on a glass plate and then dried at 80 °C for 2 h. The PPF-SSE film can be directly torn off from the glass plate after the 2 h’ drying. After that, the PPF-SSEs film was then further compacted under 100 MPa pressure to decrease the possible existing inter-particle pores. The obtained compact PPF-SSEs film are then dried at 80 °C for 2 h before vacuumed dried at 120 °C for 24 h. Then, the PAM precursor solution is prepared by dissolving 4 g acrylamide and 60 mg (NH_4_)_2_SO_4_ in 15 mL 2 M ZnSO_4_ + 0.3 M MnSO_4_ + 0.1 M KI aqueous solution. The pre-partial polymerization is conducted at room temperature for 6 h to obtain a sol solution with high viscosity. After that the viscous sol is coated on glass substrate by blade coating method for another 1 h polymerization at 40 ^o^C, which can remove free water on the sol surface and maintain its high viscosity. After that, the PPF-SSEs film is put on the partial-polymerized sol surface with certain pressure for better contact. Finally, the PPF-SSEs/PAM hydrogel asymmetric electrolyte is achieved by polymerization at 60 ^o^C for 2 h.

### The synthesis of δ-MnO_2_ cathode material

Typically, 40 mL 4 mmol of Mn(Ac)_2_·4H_2_O aqueous solution mixes with 3 mL N_2_H_4_·H_2_O (50%) drop by drop under vigorously stirring. Then, mixture solution is transferred into a 100 mL Teflon-lined stainless-steel autoclave and heated in 180 °C and maintained for 12 h. The white products are collected by centrifugation and dried at 60 °C for 12 h. After that, 0.2 g white products are dispersed in 50 mL DI water and 10 mL of NaClO solution (active chlorine >10%) is added, and the mixture is stirred for 24 h. Afterward, the δ-MnO_2_ is obtained by centrifugating, washing, and drying at 60 °C overnight.

### The synthesis of V_2_O_5_ cathode material

In detail, 0.2 mol commercial V_2_O_5_ is dissolved in 30 mL 1 M acetic acid aqueous solution under vigorously stirring for 10 h at room temperature. Then, the homogeneous solution is transferred to 50 mL Teflon-lined autoclave. The Teflon-lined autoclave heats to 200 ^o^C and is maintained at 200 ^o^C for 72 h. Finally, the products are obtained by centrifugating, washing, and drying at 40 ^o^C for 12 h.

### Computational Methods

First-principles density functional theory (DFT) calculations are performed by employing the projector-augmented wave method, as implemented in the Vienna ab initio Simulation Package (VASP) code. The exchange-correlation energy is described by the Perdew-Burke-Ernzerhof functional under the generalized gradient approximation^41^. Grimme’s DFT-D3 method is used to account for the correction from the van der Waals interactions^42^. Kinetic energy cutoff is set as 400 eV, and only gamma point was used for the Brillouin zone sampling. During the structural relaxation, the energy and force convergence criteria are set as 10^−4 ^eV and 0.05 eV·Å^−1^, respectively. In our ab initio molecular dynamics (AIMD) simulations, the Nosé-Hoover thermostat for canonical (NVT) ensemble is applied by using the VASP code^43,44^. Time step is set as 0.5 fs, and Nosé mass corresponding to 20 fs (40 time-steps) is chosen. PPFs structure is modeled by linking the Cu-coordinated porphyrin ligand with copper at the metal nodes, and the optimized lattice parameters for PPFs were *a* = 30.423 Å, *b* = 32.629 Å. During the calculations, a vacuum layer with the thickness of 15 Å is applied to avoid the spurious interaction between adjacent layers. The adsorption energy of species X on PPFs is defined as Ead(X) = E(Cu-TCPP + X) – E(Cu-TCPP) – E(X), where E(Cu-TCPP + X), E(Cu-TCPP), and E(X) represent the DFT-calculated total energy for PPFs with X adsorption, Cu-TCPP alone, and single X species, respectively. The number of charge transfer is estimated by Bader charge analysis.

### Characterization

The crystal structures are examined by a Bruker D2 Phaser X-ray diffractometer with radiation from a Cu target (*λ* = 0.154 nm) operating at 40 kV and 10 mA. The morphologies are examined by field emission scanning electron microscopy (FESEM; JEOL JSM-6700F, 5 kV). The chemical state and composition are investigated by an ESCALAB 250 photoelectron spectroscopy (Thermo Fisher Scienctific) at 1.2 × 10^−9^ mbar using Al Kα X-ray beam (1486.6 eV). All XPS spectra are calibrated by shifting the detected adventitious carbon C 1 s peak to 284.4 eV. The in situ hydrogen fluxes are detected by the airtight battery system equipped with gas chromatography-mass spectrometry. In the battery-gas chromatography quantitative analysis measurement, the airtight battery system is connected to a gas chromatography (Agilent GC-2014). The Zn anode surface after electrochemical reactions, are analyzed utilizing a combination of Raman and Ar Plasma sputtering techniques. In details, the Zn anode is etched by Ar sputtering technique. After etching, the Zn anode is exposed, and the component analysis is conducted by Raman measurement. The chemical coordinated information is obtained by X-ray absorption fine spectroscopy (XAFS) spectra conducted at the beamline BL11B1 of Shanghai Synchrotron Radiation Facility (SSRF) at Shanghai Institute of Applied Physics, Chinese Academy of Sciences.

### Electrochemical tests

In a typical process, the active materials are mixed with Ketjen Black and polyvinylidene fluoride (PVDF) in a 95:5:5 weight ratio. Then, the mixture is dispersed in a small methyl-2-pyrrolidinone (NMP) solvent by grinding in an agate mortar to achieve a stably homogeneous ink. After that, the ink is pasted on carbon fiber cloth (CFC) and dried at 80 ^o^C for 12 h in a vacuum. The battery is assembled using active materials modified CFC as cathode, the as-prepared electrolyte and metallic Zn foil with anode-electrolyte-cathode set. Galvanostatic cycling studies are performed using LAND battery testing system (CT2001A) at room temperature. The cyclic voltammetry (CV) and linear scan voltammetry (LSV) are carried out utilizing CHI 760e electrochemical workstation (CHI 760 D, Chenhua, Shanghai).

To evaluate the electrochemical Zn stripping/plating behavior and cycling stability of the bare Zn electrode, the symmetric cells are assembled in standard CR2032-type coin cell using PAM hydrogel and as-designed asymmetric electrolytes. The ionic conductivities are conducted by SS plate with two electrode configuration and calculated according to the following equation:

$$\rho=(R\times A)/d$$

where *ρ* is the resistivity of ionic conductor, *R* is the resistance, *A* is the area of ionic conductor, and *d* is the thickness of the ionic conductor, respectively.

## Supplementary information

Supplementary Information

## Data Availability

The data that support the findings of this study are available within the text including the Methods, and Supplementary information. Raw datasets are available from the corresponding author upon reasonable request.
