# Supplementary material for: An asymmetric electrolyte to simultaneously meet contradictory requirements of anode and cathode
Source: Nat Commun. 2023 May 22;14:2925. doi: 10.1038/s41467-023-38492-8 (PMC10202929; doi:10.1038/s41467-023-38492-8)
Supplement: Supplementary file 1 — Supplementary Information [file 41467_2023_38492_MOESM1_ESM.pdf]

Supporting Information

An Asymmetric Electrolyte to Simultaneously Meet Contradictory Requirements of Anode and Cathode

Shengmei Chen[1], Yiran Ying[2], Longtao Ma[1], Daming Zhu[3], Haitao Huang[2], Li, Song[4], Chunyi Zhi[1, 5]*

[1] Department of Materials Science and Engineering, City University of Hong Kong 83 Tat Chee Avenue, Kowloon, Hong Kong 999077, P. R. China.

[2] Department of Applied Physics and Research Institute for Smart Energy, The Hong Kong Polytechnic University, Hung Hom, Kowloon, Hong Kong 999077, P. R. China.

[3] Shanghai Synchrotron Radiation Facility, Shanghai Advanced Research Institute, Chinese Academy of Sciences, Shanghai 201204, P. R. China.

[4] National Synchrotron Radiation Laboratory, CAS Center for Excellence in Nanoscience, University of Science and Technology of China, Hefei, 230029, P. R. China.

[5] Hong Kong Institute for Clean Energy, City University of Hong Kong, Kowloon 999077, Hong Kong, P. R. China.

Corresponding author: Prof. Chunyi Zhi, Email: cy.zhi@cityu.edu.hk

S. Chen, Y. Ying and L. Ma equally contribute to this work.

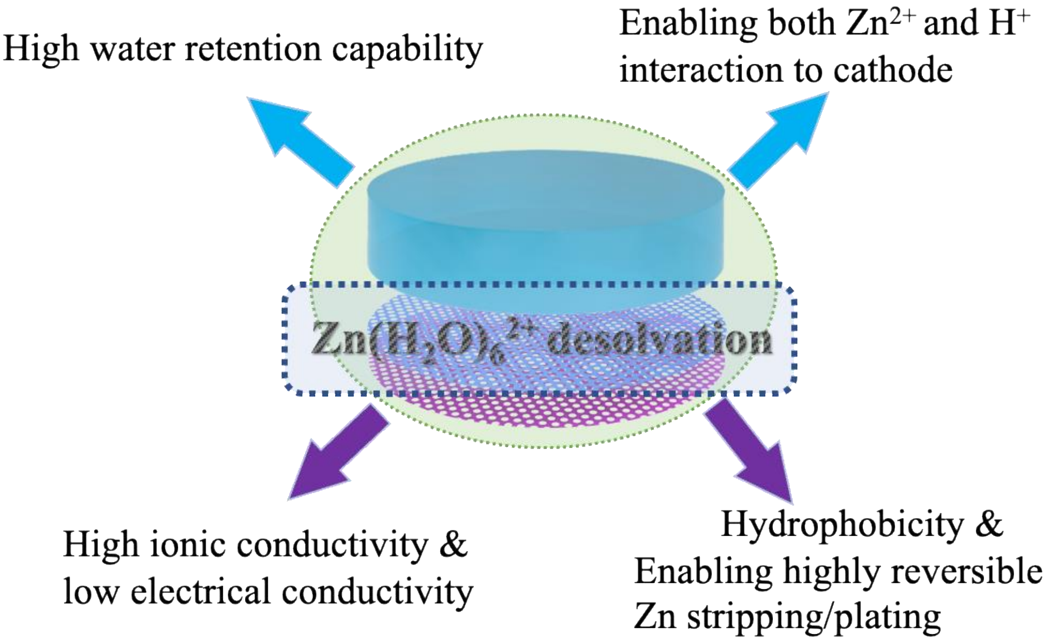

Supplementary Fig. 1 | Design strategies of asymmetric 2D PPF-SSEs/PAM hydrogel electrolyte for metal-ion batteries. The hydrogel electrolyte with super-absorbency can contain vast electrolyte solutions, while the PPF-SSEs can preclude H_2O molecules encountering Zn metal electrode.

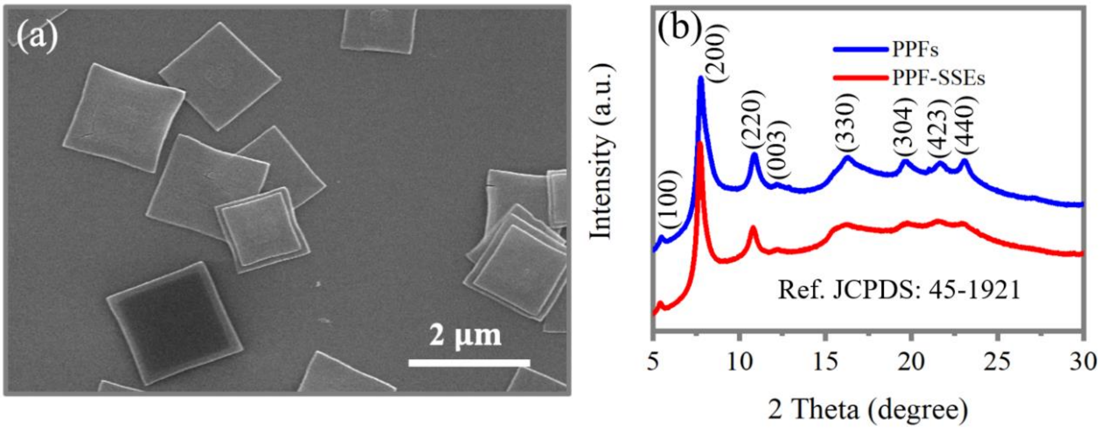

Supplementary Fig. 2 | SEM and XRD Characterizations of PPFs and PPF-SSEs. (a) SEM image of PPFs. (b) XRD spectra of PPFs and PPF-SSEs. The typical peaks of PPF nanosheets in the XRD patterns indicate a tetragonal structure with good crystallinity. We index the XRD pattern of the PPF crystal based on the crystal structure ref. no. # 45-1921 in the JCPDS.

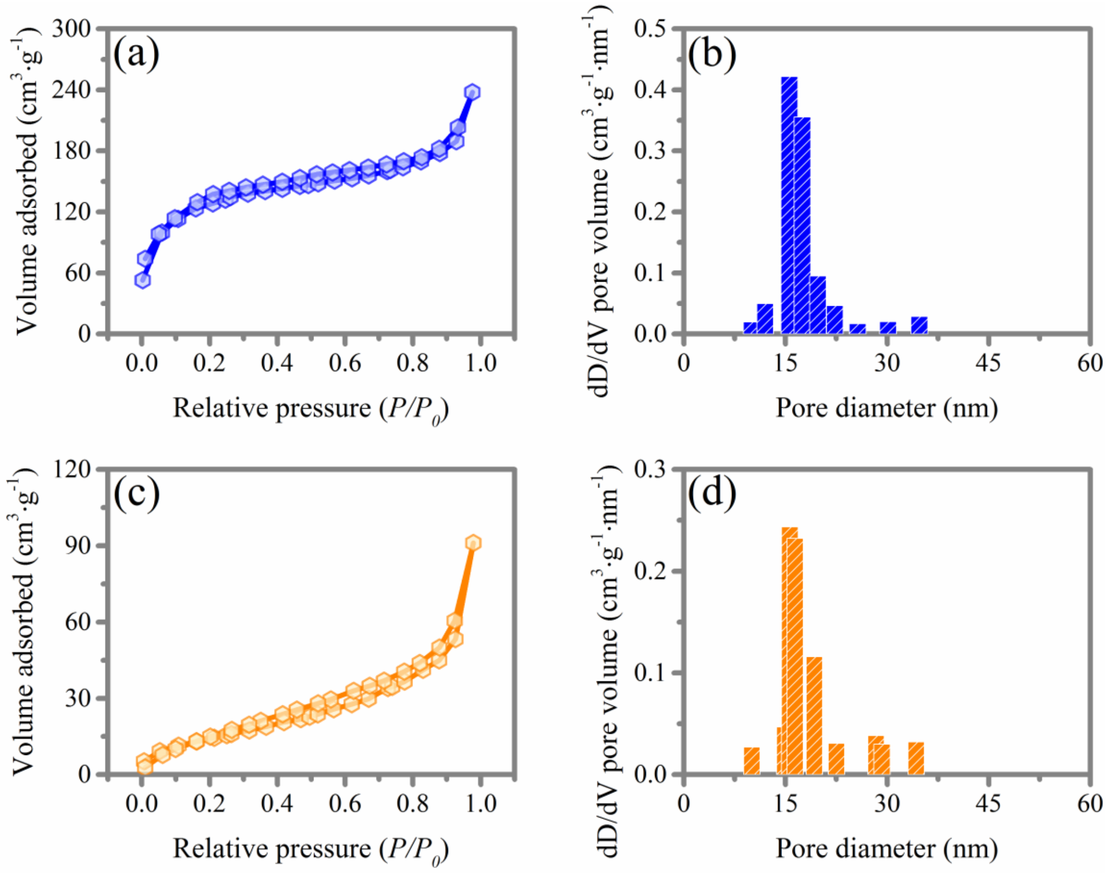

Supplementary Fig. 3 | N_2 adsorption/desorption isothermal and BJH pore size distribution of PPFs and PPF-SSEs. 77 K N_2 adsorption/desorption isothermal of (a) PPFs and (c) PPF-SSEs, and BJH pore size distribution of (b) PPFs and (d) PPF-SSEs.

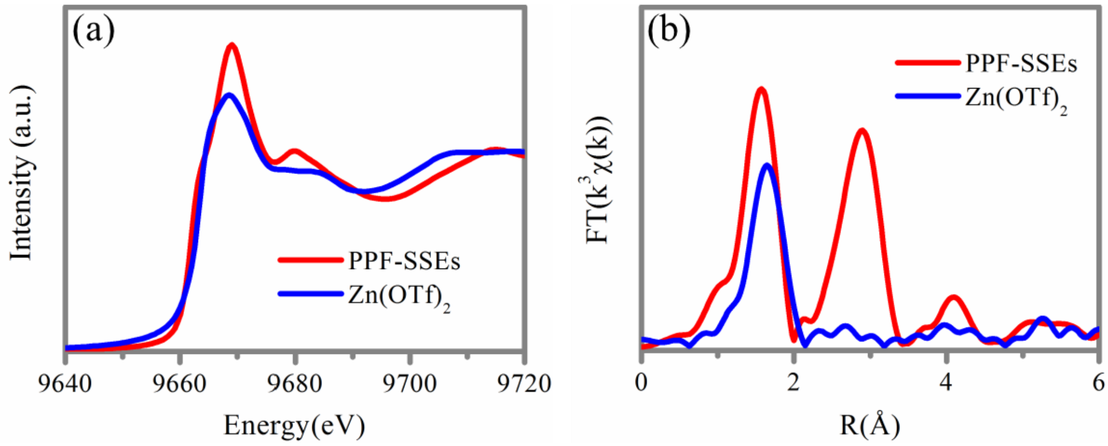

Supplementary Fig. 4 | XANES and Fourier-transformed EXAFS spectra of PPF-SSEs and $Zn(OTf)_2$. (a) Zn K-edge XANES spectra and (b) Fourier-transformed EXAFS spectra collected at the Zn K-edge of PPF-SSEs and $Zn(OTf)_2$.

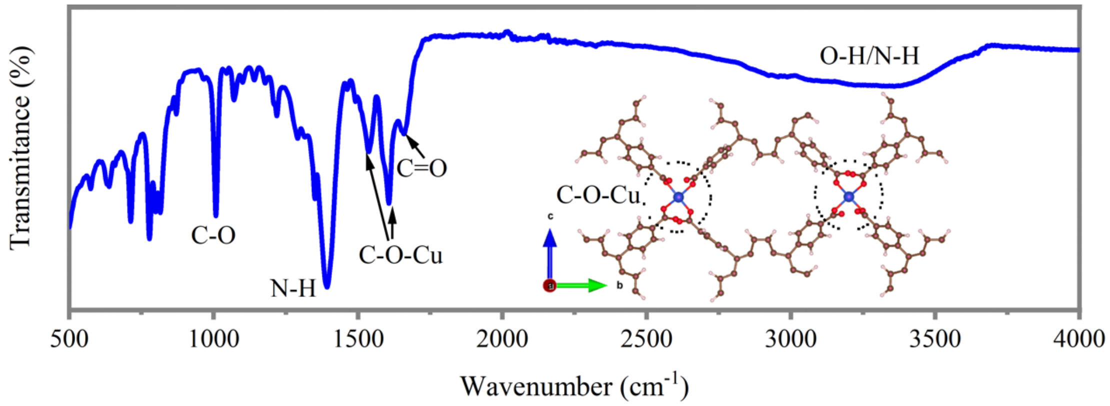

Supplementary Fig. 5 | FT-IR spectra of PPFs. The inset is the crystal structure of PPFs.

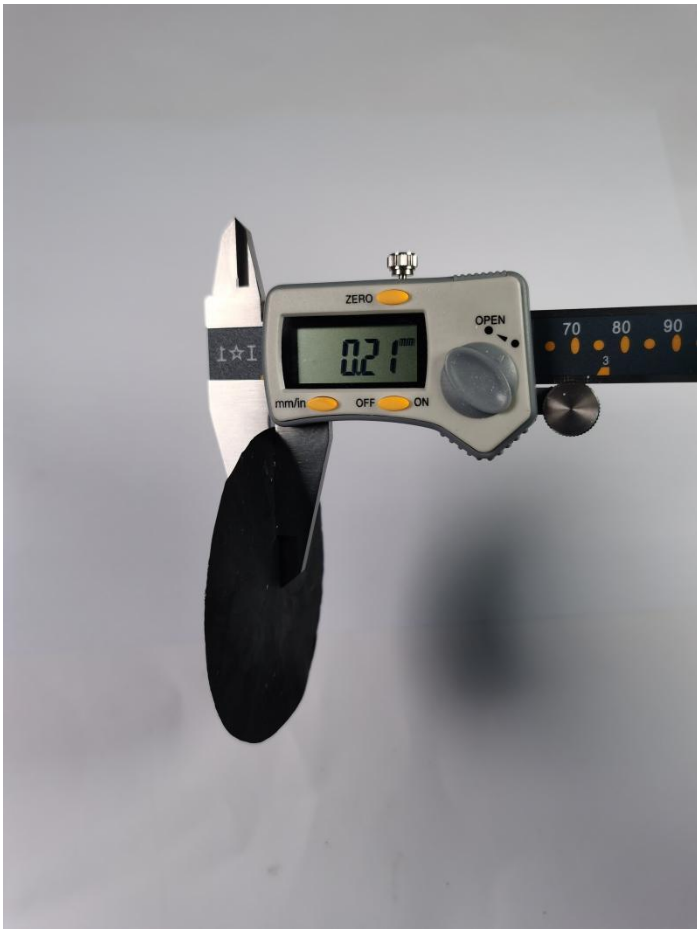

Supplementary Fig. 6 | Photograph of asymmetric electrolyte to characterize its thickness.

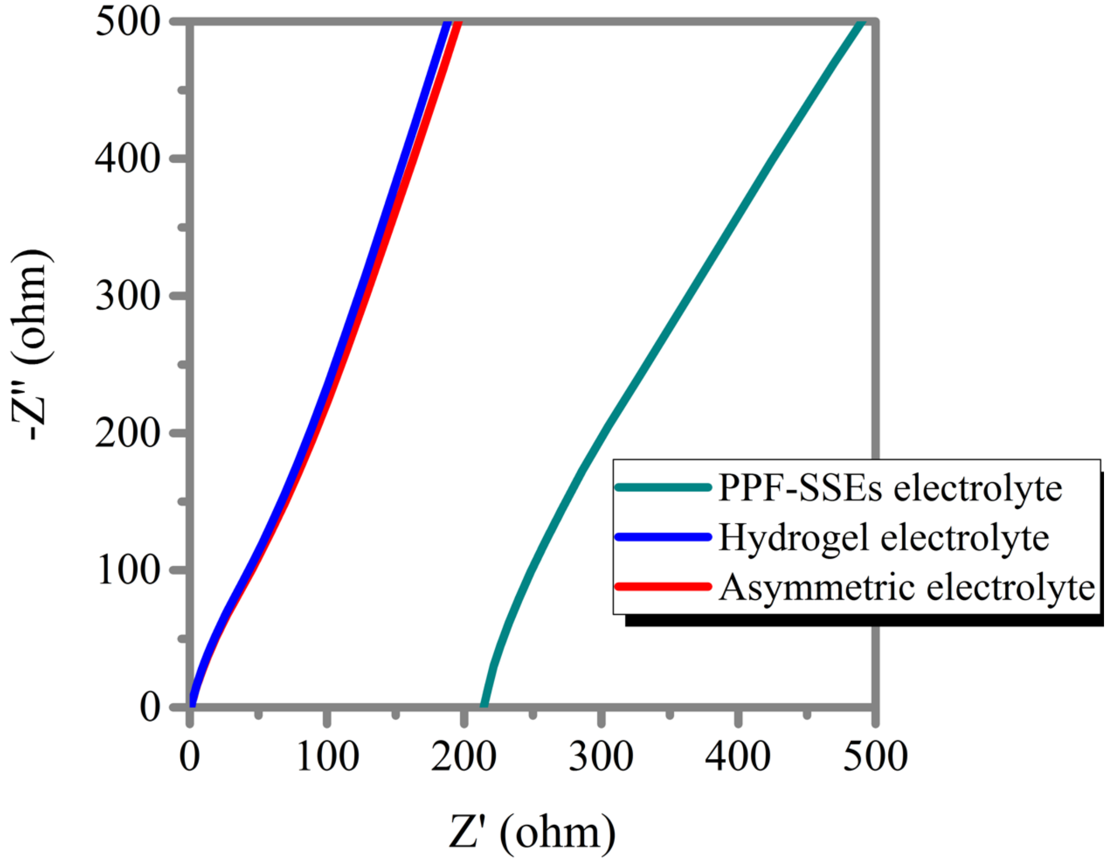

Supplementary Fig. 7 | The A. C. impedance spectra to determine the ionic conductivity of PPF-SSEs, hydrogel electrolyte and asymmetric electrolyte.

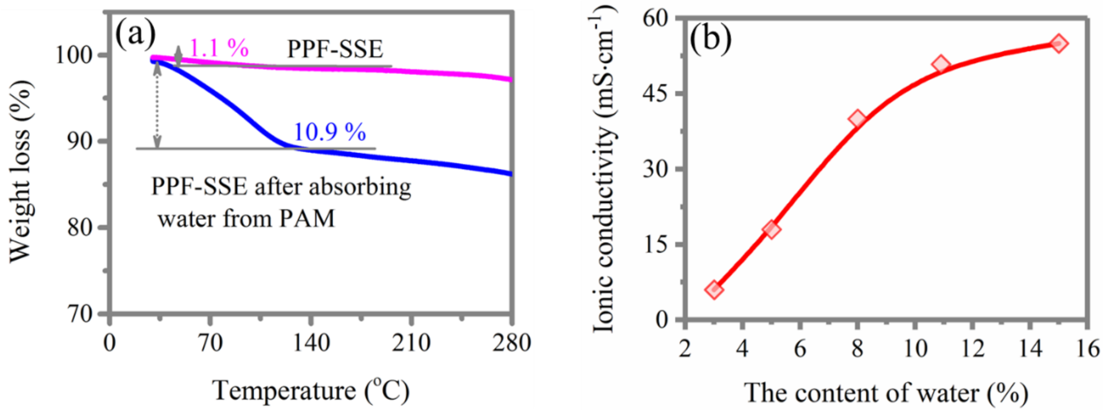

Supplementary Fig. 8 | Thermogravimetric analysis and ionic conductivity of PPF-SSE. (a) Thermogravimetric analysis for evaluating the trace amount of water in the PPF-SSE. (b) The ionic conductivity of PPF-SSE changes with the increase of water content.

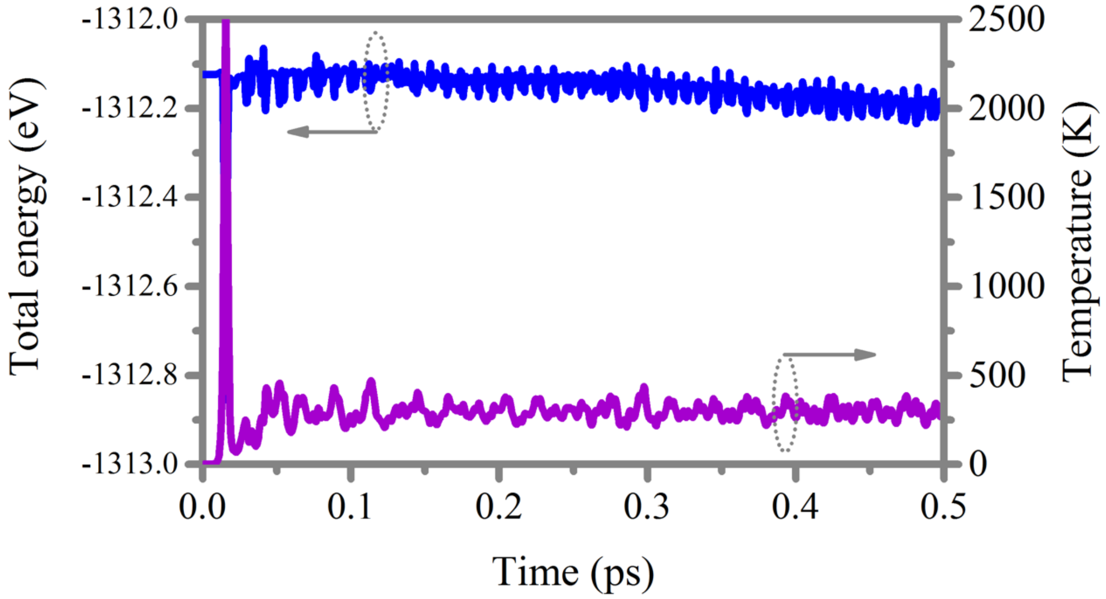

Supplementary Fig. 9 | Total energy (blue) and temperature (purple) of the system as a function of time in the AIMD simulation.

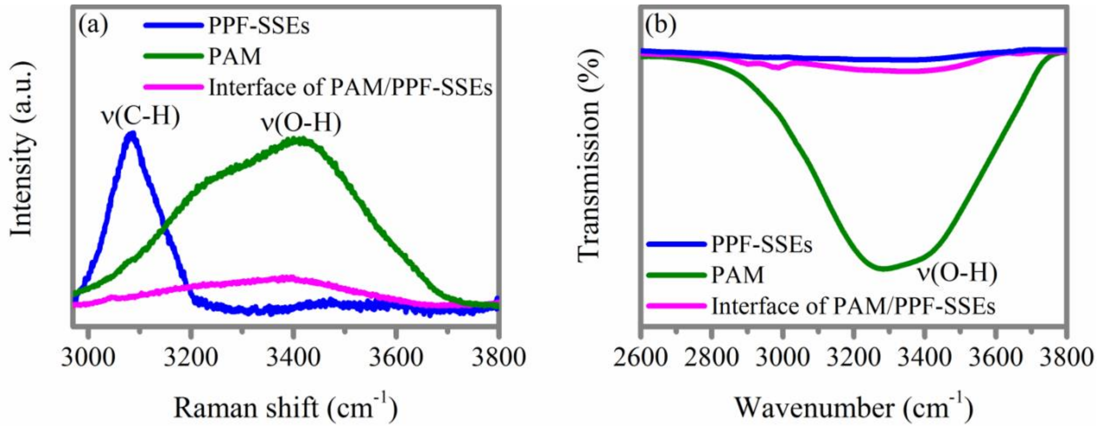

Supplementary Fig. 10 | Raman and FT-IR spectra of various electrolytes. (a) Raman spectra and (b) FT-IR spectra of PPF-SSEs, PAM and interface of PAM/PPF-SSEs.

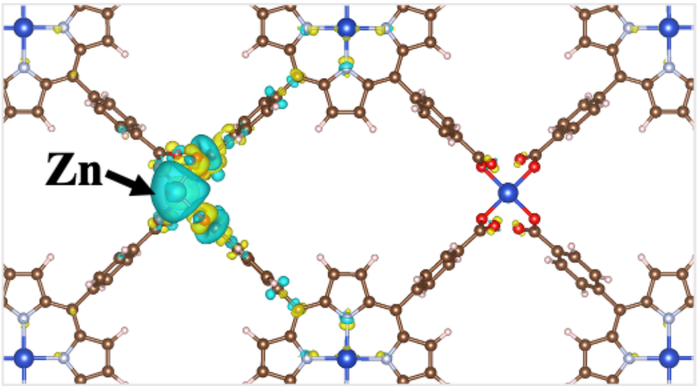

Supplementary Fig. 11 | Differential charge density distribution of PPF with Zn^{2+} adsorption.

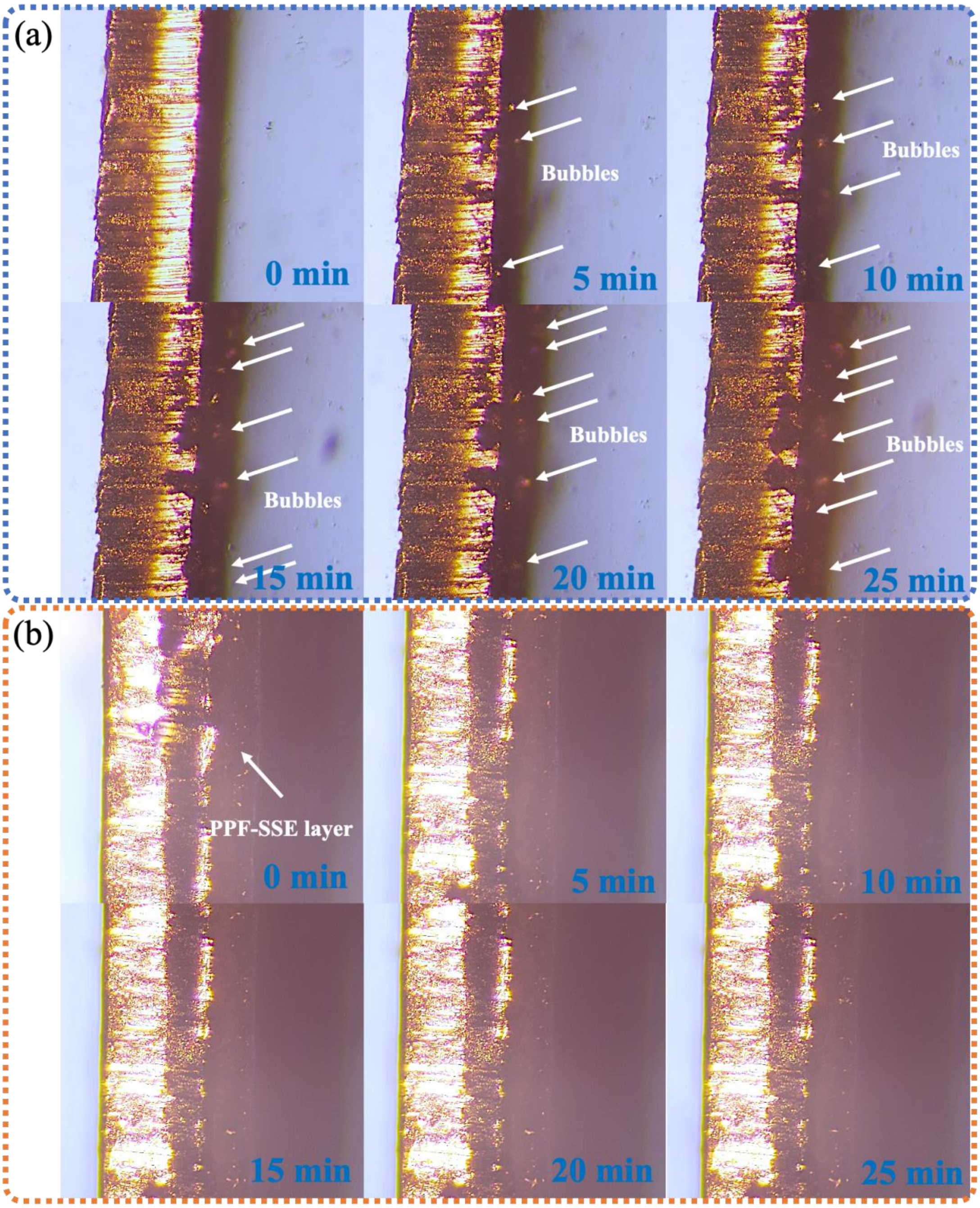

Supplementary Fig. 12 | *In-situ* optical visualization observations of Zn plating behavior. *In-situ* optical visualization observations of Zn plating behavior of bare Zn foil (a) and Zn with PPF-SSEs (b).

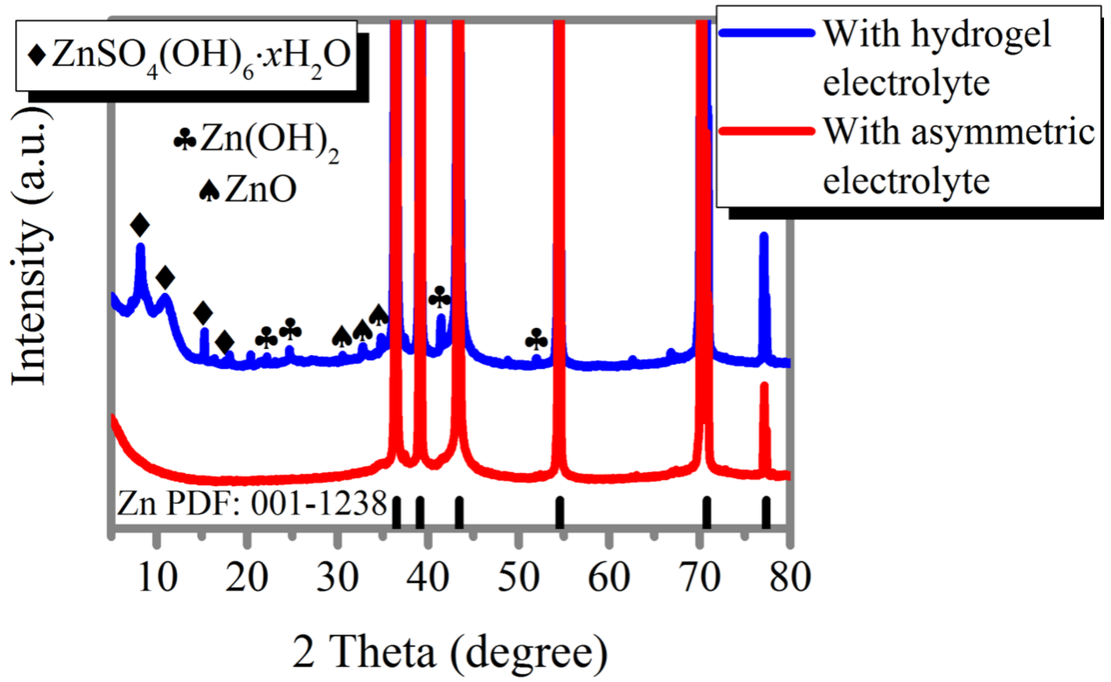

Supplementary Fig. 13 | XRD analysis of Zn foil with hydrogel electrolyte and asymmetric electrolyte after 10 cycles.

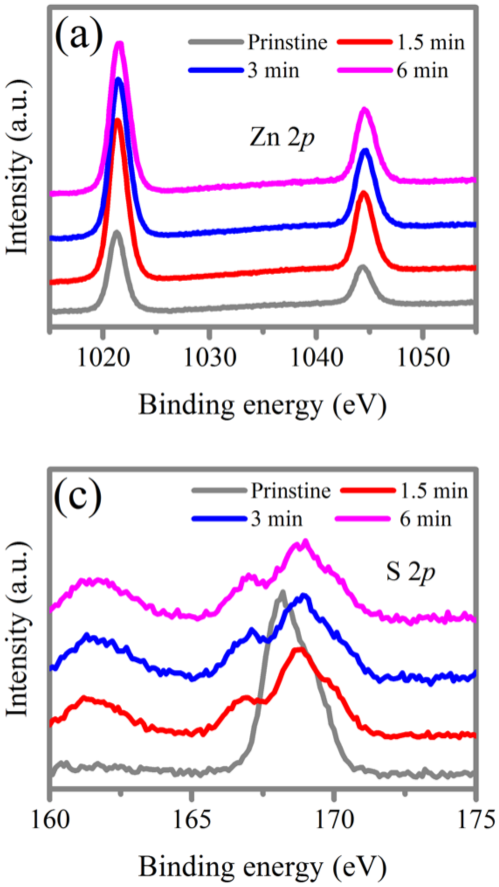

Supplementary Fig. 14 | Depth profiles of high resolution XPS spectra of deposited Zn using PAM hydrogel electrolyte. (a) Zn 2*p*, (b) O 1*s* and (c) S 2*s* after 0 min, 1.5 min, 3 min, 6 min Ar^+ sputtering to characterize the composition of deposited Zn using PAM hydrogel electrolyte.

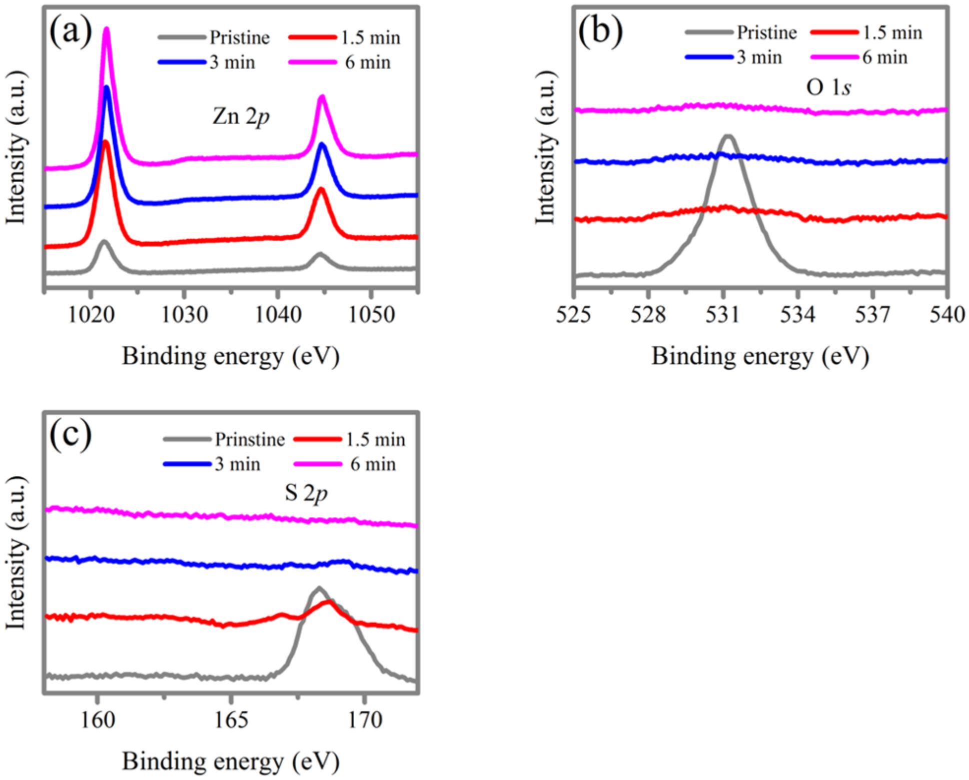

Supplementary Fig. 15 | Depth profiles of high resolution XPS spectra of deposited Zn using PPF-SSEs/hydrogel/PPF-SSEs electrolyte. Depth profiles of high resolution XPS spectra for (a) Zn 2*p*, (b) O 1*s* and (c) S 2*s* after 0 min, 1.5 min, 3 min, 6 min Ar^+ sputtering to characterize the composition of deposited Zn using PPF-SSEs/hydrogel/PPF-SSEs electrolyte.

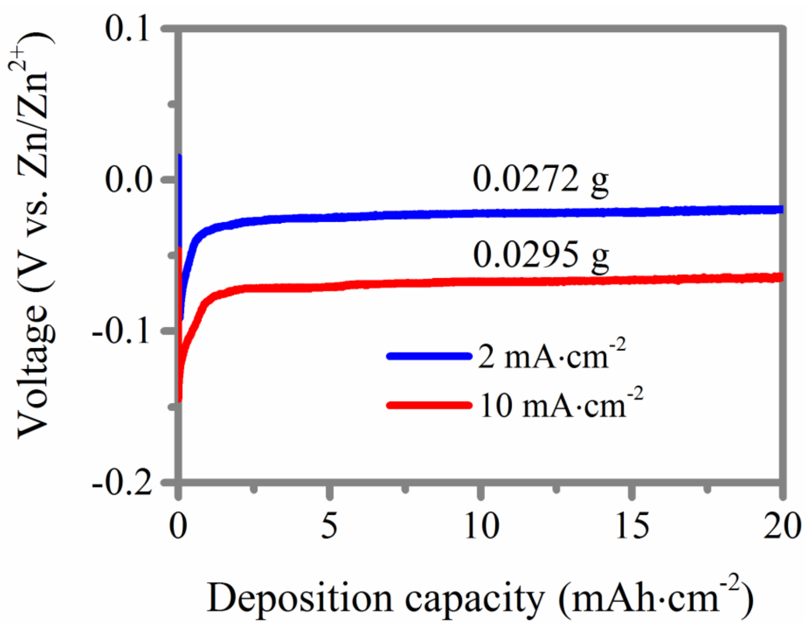

Supplementary Fig. 16 | The Zn//Cu cells are used to evaluate real specific capacity of Zn dissolution.

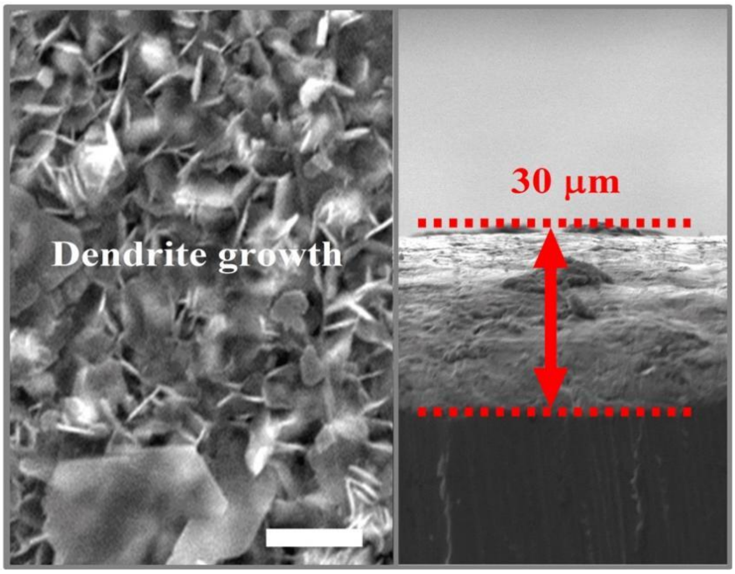

Supplementary Fig. 17 | SEM images and cross-section SEM images of Zn anode harvested from Zn//Zn cell using PAM hydrogel electrolyte. Scale bar: 5 μm.

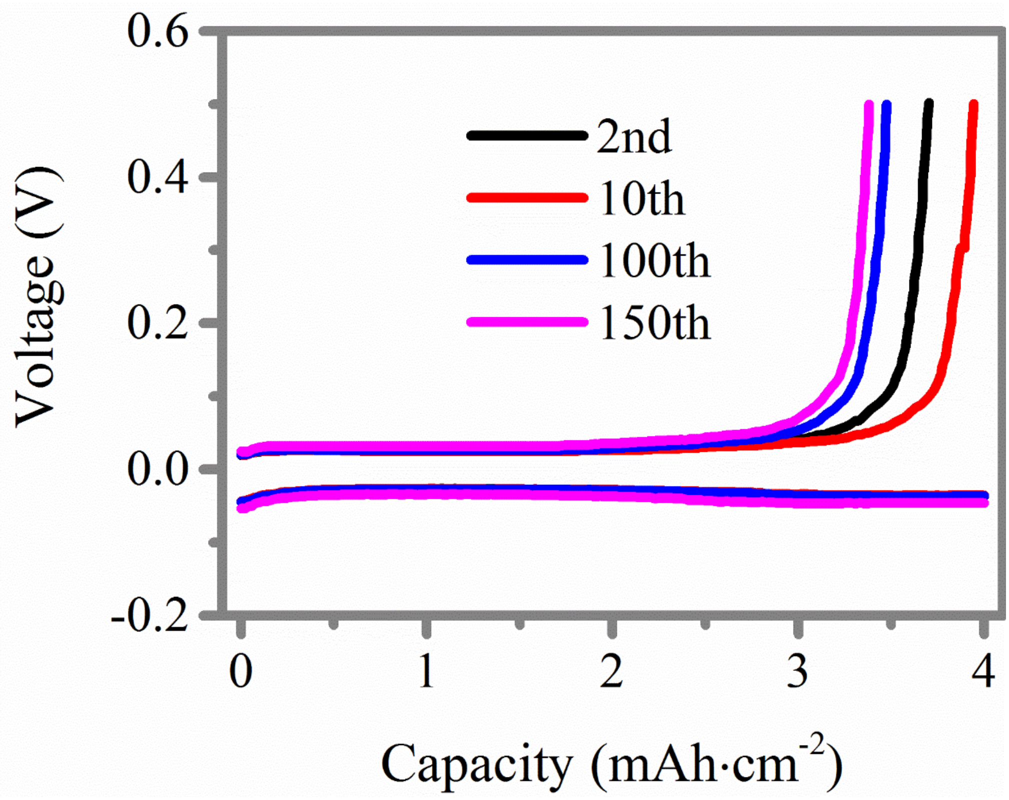

Supplementary Fig. 18 | Zn deposition/dissolution profiles on Cu electrode cycled in the PAM hydrogel electrolyte.

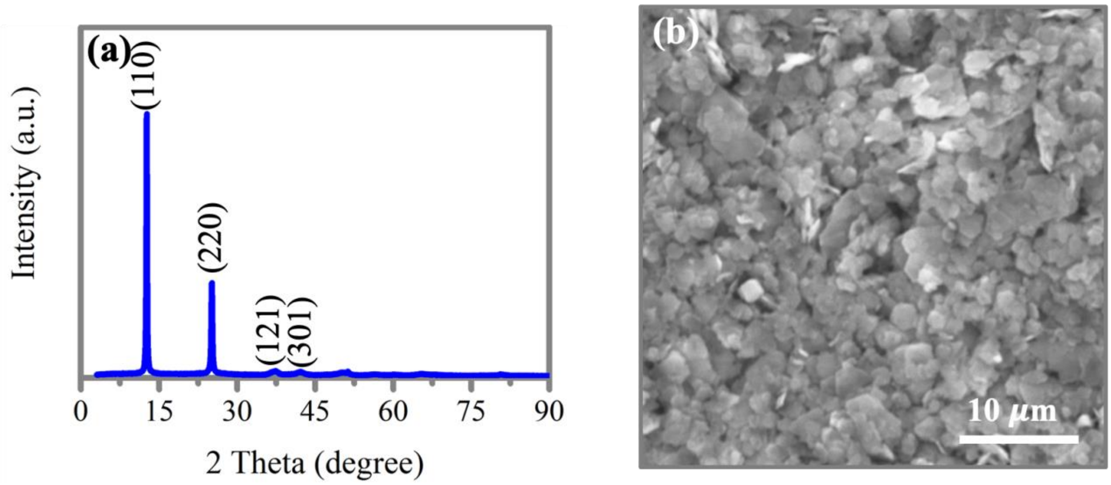

Supplementary Fig. 19 | The XRD pattern and SEM image of the δ-MnO_2.

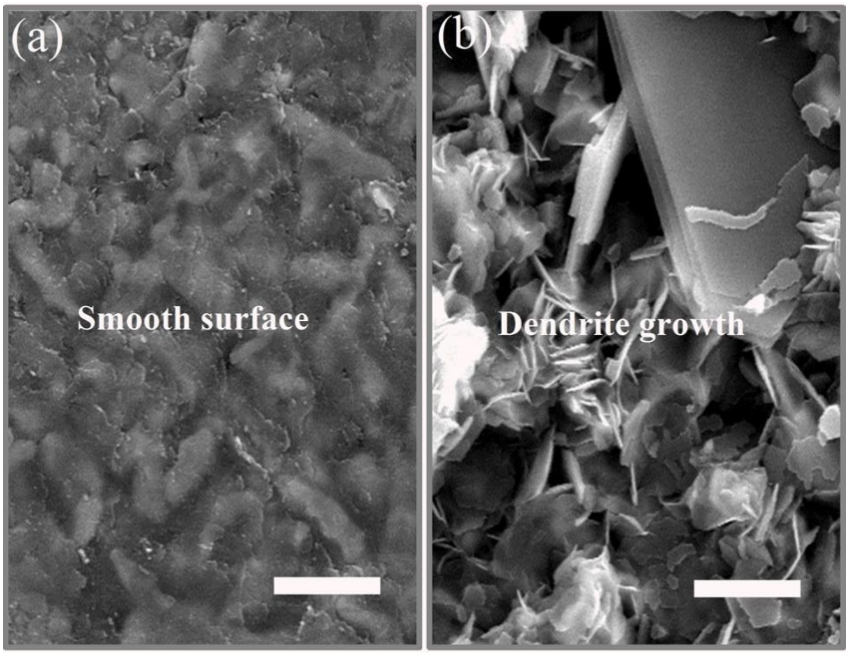

Supplementary Fig. 20 | SEM images of Zn anode harvested from Zn//MnO_2 full cell after cyclic test with various electrolytes. (a) asymmetric electrolyte and (b) PAM hydrogel electrolyte. Scale bar: 5 μm.

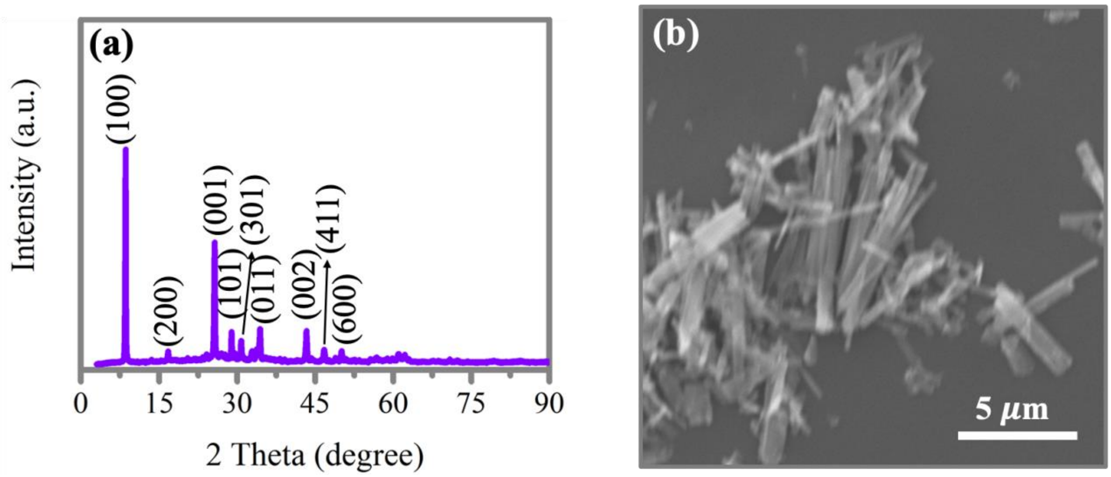

Supplementary Fig. 21 | The XRD pattern and SEM image of V_2O_5.

Supplementary Table 1 | The Zn K-edge EXAFS curve fitting parameters for PPF-SSEs and $Zn(OTf)_2$.

Samples	Coordination number	Bond length	$\sigma^2 (10^{-3}$ Å$^2)$
PPF-SSEs	3.7	1.96	6.2
$Zn(OTf)_2$	2.9	2.07	6.1

Note, compared with $Zn(OTf)_2$, the Zn K-edge absorption shifts to low energy, demonstrating an decreased in Zn oxidation state (Supplementary Fig. 4a). Meanwhile, Fourier-transformed EXAFS analysis reveals the appearance of Zn-Zn scattering signal, suggesting the formation of a new Zn-Zn coordination shell (Supplementary Fig. 4b). The increased coordination number of PPF-SSEs may originate from the interactions between infilled Zn^{2+} with PPF framework.

Supplementary Table 2 | The parameters of high capacity Zn//MnO_2 pouch-type cell.

**Parameters**	
Capacity	1100 mAh
MnO_2 mass loading	15.23 mg·cm^{-2}
Active area	(7.5*8.5)*2*3=382.5 cm^2
Areal capacity	2.88 mAh·cm^{-2}
Total MnO_2 mass	5.83 g
Carbon cloth fiber	~ 250 μm
Zn foil	~ 200 μm
Electrolyte	210 μm
Package + Tabs	3.81 g
Pouch cell thickness	3.42 mm
Pouch cell weight	35.69 g
Energy density	40.98 Wh·kg^{-1}
